# Supplementary material for: Placental Complement Activation in Fetal and Neonatal Alloimmune Thrombocytopenia: An Observational Study
Source: Int J Mol Sci. 2021 Jun 23;22(13):6763. doi: 10.3390/ijms22136763 (PMC8267834; doi:10.3390/ijms22136763)
Supplement: Supplementary file 1 [file ijms-22-06763-s001.zip › ijms-1233948-supplementary.pdf]

Supplementary Materials:

Placental Complement Activation in Fetal and Neonatal Alloimmune Thrombocytopenia: An Observational Study

Supplemental Figure S1 – Semi quantitative scoring complement depositions

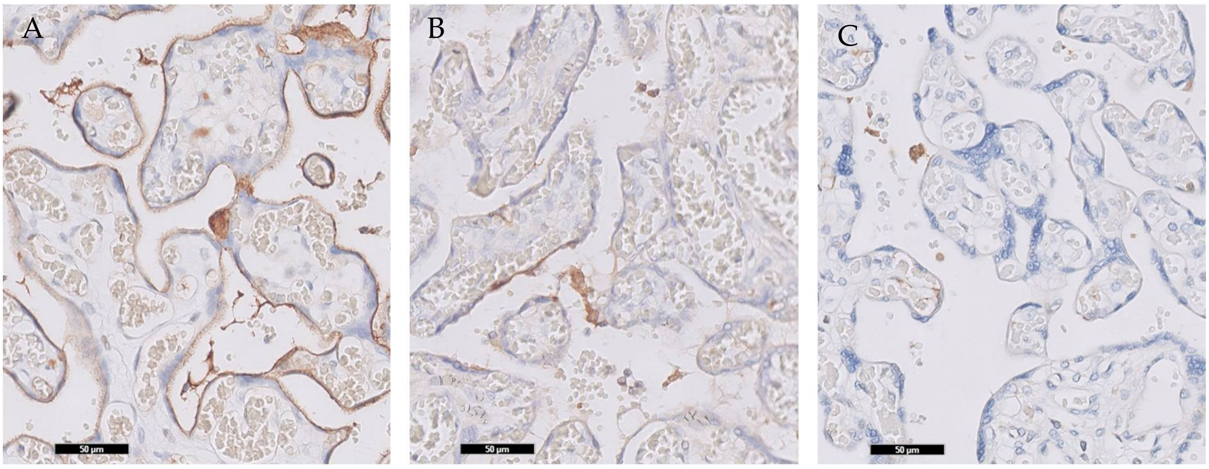

Sections of placentas stained immunohistochemically for C4d, **A** shows diffuse deposition of C4d to the syncytiotrophoblast, in **B** focal binding to syncytiotrophoblast of C4d appreciated and **C** shows an example where C4d binding to syncytiotrophoblast was absent.

Supplemental Figure S2 – Semi quantitative scoring of complement deposition fetal vessels

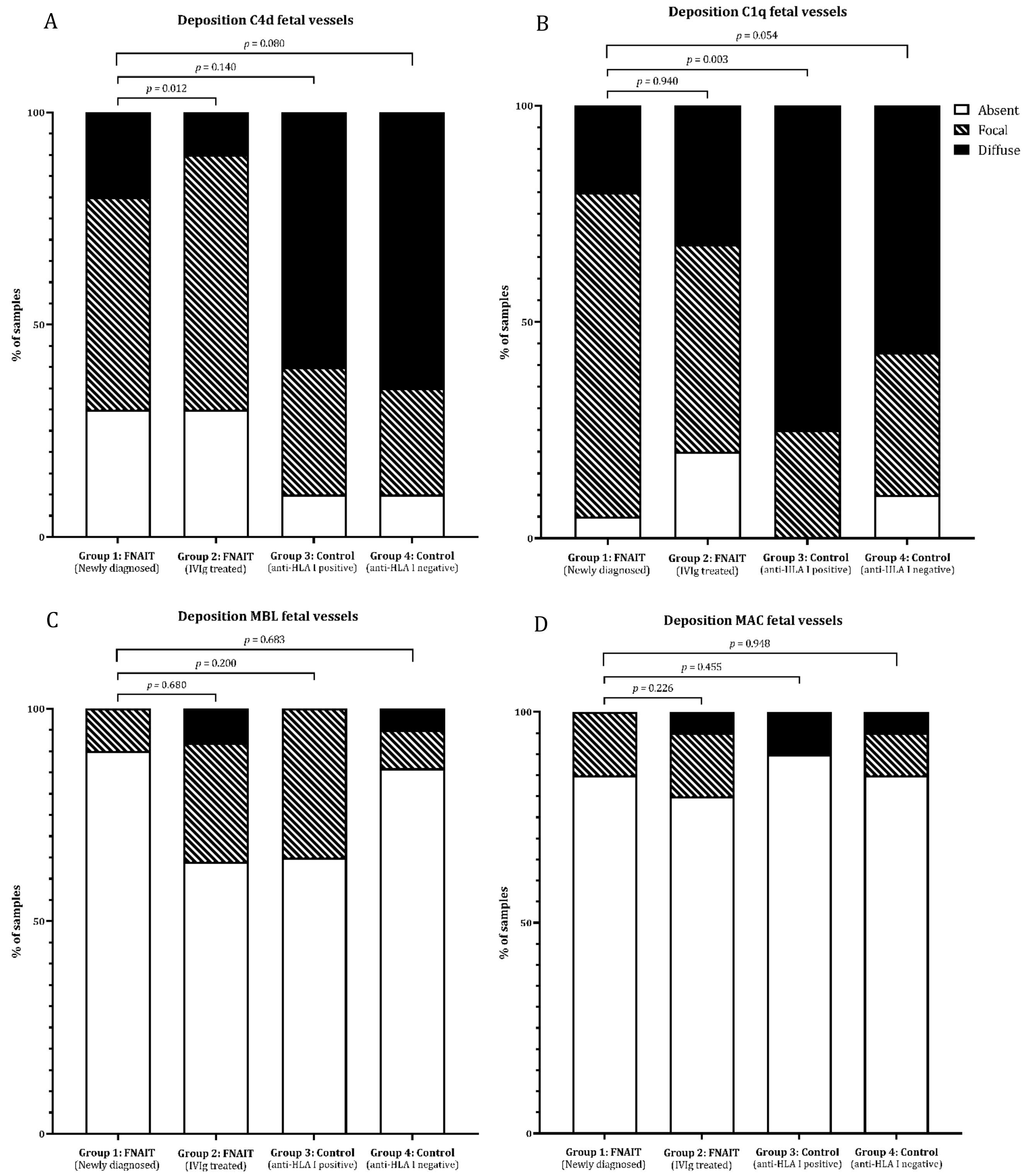

All complement depositions shown by immunohistochemistry in the fetal vessels were scored semi-quantitatively as absent (<10%), focal (10-50%) or diffuse (>50%). **Figure (A)** summarizes the scoring of C4d deposition at the fetal vessels, in **Figure (B)** scoring of C1q deposition, in **Figure (C)** scoring of MBL deposition and **Figure (D)** scoring of MAC deposition are summarized. Ordinal logistic regression was used to compare complement deposition score between the groups.

FNAIT, fetal neonatal alloimmune thrombocytopenia; IVIg, intravenous immune globulins; HLA, human leukocyte antigen; MBL, mannose-binding lectin; MAC, membrane attack complex.

Supplemental Figure S3 –Complement deposition in the placenta

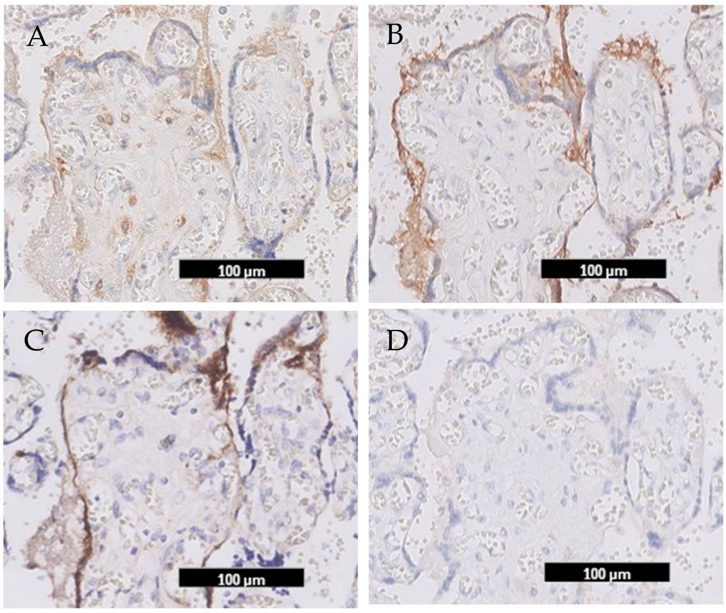

Sequential sections of placentas stained immunohistochemically for components of the complement system. **A** shows diffuse deposition of C1q to the syncytiotrophoblast, in **B** diffuse binding of C4d to syncytiotrophoblast can be seen and **C** shows an example of binding of (MAC) SC5b-9. Figure **D** shows that the mannose-binding lectin (MBL) is absent.

Supplemental Table S1 – Clinical data and immunohistopathology per case

Case		Age mother	G/P	GA	αHPA	αHLA	Sex	SGA	Bleed	Plt.	C1q syn	C4d syn.	MAC syn.	MBL syn.	C1q f.v.	C4d f.v.	MAC f.v.	MBL f.v	PW p<10	Maturation	MVM	FVM	Infection	VUE	Fetal hypoxia
Newly diagnosed FNAIT	1	34	G1P0	41+0	1a	No	F	No	Skin	44	D/F	F/D	A/F	A/F	F/F	A/A	A/A	A/F	No	Delayed	Infarct	-	-	-	-
	2	35	G2P0	40+1	1a	Yes*	M	Yes	None	17	D/D	D/D	D/A	A/A	A/F	A/A	A/A	A/F	Yes	Corresp. GA	-	AV	-	Low	-
	5	28	G2P1	37+1	1a+5b	Yes	M	Yes	Skin	5	D/D	D/D	A/A	A/A	F/F	F/D	A/F	A/A	No	Corresp. GA	-	-	-	-	-
	7a	30	G3P1	32+2	1a	Yes	F	NT	ICH	11	D	A	D	A	F	F	A	A	No	Delayed	-	-	-	-	-
	11	33	G1P0	34+3	1a+3a	No	M	No	Skin	41	A	A	D	A	F	F	F	A	Yes	Accelerated	Infarct abrupt	-	Stage 1	-	Mild
	12	31	G2P1	38+3	1a	Yes	F	Yes	Skin	10	D	F	A	A	D	F	A	A	Yes	Delayed	-	-	-	Low	-
	13	32	G1P0	35+1	1a	No	M	No	Skin	40	F/D	A/F	A/A	A/A	D/F	D/D	A/A	A/A	No	Corresp. GA	DVH	-	-	-	-
	14	21	G2P0	41+4	1a	Yes*	M	No	ICH	8	F/F	A/F	F/F	A/A	D/F	D/F	A/A	A/A	No	Delayed	-	-	Stage 1	-	-
	15	29	G2P1	32+6	1a+5b	Yes	F	Yes	None	68	F	D	F	A	F	F	A	A	Yes	Accelerated	Infarct	-	-	Low	Mild
FNAIT (IVIg)	7b	32	G4P2	35+5	1a	Yes	F	No	None	22	F/D	A/A	F/F	A/A	F/D	F/F	A/F	A/A	No	Corresp. GA	-	-	-	-	-
	8	31	G3P2	38+1	1a+3a	Yes	M	No	None	266	D	A	A	-	D	D	A	-	No	Delayed	-	-	-	Low	-
	11	35	G3P1	37+2	1a	No	F	No	None	169	F	A	A	A	F	A	F	A	Yes	Corresp. GA	-	-	-	-	-
	37	37	G2P1	38+0	1a	Yes	M	No	None	78	D	A	D	A	A	F	A	A	NT	Corresp. GA	DVH	-	-	-	-
	38	31	G5P1	39+2	1a	No	M	Yes	None	50	A	A	D	A	F	F	D	A	No	Corresp. GA	-	-	-	-	-
	39	33	G2P1	38+5	1a	NT	M	No	None	30	D	F	A	A	A	A	A	A	No	Corresp. GA	-	-	Stage 1	-	-
	40	33	G2P1	39+0	1a	No	M	No	None	85	F	A	D	A	A	F	A	A	NT	Delayed	IS	-	-	Low	-
	41	31	G3P1	38+6	1a	No	M	No	None	18	F/F	A/A	A/F	A/-	D/F	F/F	A/A	A/-	NT	Delayed	-	-	-	-	-
	42	30	G2P1	38+4	1a	Yes*	M	No	None	24	A/F	F/A	F/D	A/A	F/F	F/F	F/A	F/F	NT	Corresp. GA	-	-	-	-	-
	43	35	G5P2	38+5	1a	No	F	No	None	8	F	A	A	A	F	F	A	A	NT	Delayed	-	-	-	Low	-
	46	38	G3P2	37+6	1a	Yes*	F	No	None	271	A/F	A/A	D/D	A/A	D/D	D/F	A/A	F/F	No	Corresp. GA	-	-	-	-	Mild
	47	30	G4P1	36+0	1a	Yes*	F	No	ICH	6	-/A	A/A	D/D	A/A	-/F	A/F	A/A	F/F	No	Delayed	DVH	-	-	-	-
	48	37	G4P2	39+2	1a	NT	F	No	None	172	A/A	A/A	D/D	A/A	A/F	A/F	A/A	A/F	No	Corresp. GA	DVH, IS	-	-	-	-
	50	26	G2P1	37+0	1a	Yes	F	No	None	164	A	A	A	A	A	A	A	A	No	Corresp. GA	-	-	-	-	-

G, gravidity; P, parity; GA, gestational age; αHPA, anti-human platelet antigen directed antibodies; αHLA, anti-fhuman leukocyte class I antibodies; SGA, small for gestational age; Bleed, bleeding symptoms; Plt, lowest platelet count; syn., syncytiotrophoblast; MAC, membrane attack complex; MBL, mannose binding lectin; f.v. fetal vessels; PW, placenta weight; MVM, maternal vascular malperfusion; FVM, fetal vascular malperfusion; VUE, villitis of unknown origin; F, focal; D, diffuse; A, absent; *Specificity of antibodies could not be determined; Corresp. GA, corresponding with gestational age; NT, not tested; ICH, intracranial haemorrhage; AV, avascular villi; Low, low grade focal; DVH, ditstal villous hypoplasia, IS, ischemia.

Supplemental Table S2 – Immunohistochemistry protocols

	C1q	C4d	MAC (SC5b-9)	MBL
**Material type**	Sections of parafin embedded placenta			
**Preparation/fixation**	Deparaffinize			
**Antigen retrieval**	Heat antigen retrieval (Tris/EDTA, 10 mM, pH 9.0)	Heat antigen retrieval (Tris/EDTA, 10 mM, pH 9.0)	Enzym antigen retrieval, Prot 24 (XXIV) (Sigma Aldrich, Saint Louis, Missouri, USA, P8038-50MG)	Heat antigen retrieval (Citric acid, 10mM, pH 6.0)
**Block endogenous peroxidase**	H2O2 (30%) was used 1:250 in distilled water (Merck, Kenilworth, New Jersey, United States of America, 1.07209.0250)			
**Block**	NGS 1:20 in PBS/1% BSA (Normal goat serum, DakoCytomation, Glostrup. Denmark, X0907)			
**Primary antibody**	anti-C1q, Rabbit, HRP conjugated, 1:2000 (DakoCytomation, Glostrup. Denmark, A0138)	anti-C4d, Rabbit, HRP conjugated, 1:75 (Biomedica, Vienna, Austria, BI-RCD4D)	anti-SC5b-9 (neoantigen), Mouse, HRP conjugated, 1:1000 (Quidel Corporation, San Diego, California, USA, A239)	anti-MBL, Rabbit, HRP conjugated 1:500 (Sigma Aldrich, Saint Louis, Missouri, USA HPA002027)
**Incubation primary antibody**	60 minutes	60 minutes	Overnight	60 minutes
**Isotype specific control (negative control)**	Rabbit negative control fraction, (DakoCytomation, Glostrup. Denmark, X0936)	Rabbit negative control fraction, (DakoCytomation, Glostrup. Denmark, X0936)	Mouse negative control, IgG2b (DakoCytomation, Glostrup. Denmark, X0944)	Rabbit negative control fraction, (DakoCytomation, Glostrup. Denmark, X0936)
**Secondary antibody**	HRP anti-rabbit envision+ system (DakoCytomation, Glostrup. Denmark, K4003)	HRP anti-rabbit envision+ system (DakoCytomation, Glostrup. Denmark, K4003)	HRP anti-mouse envision+ system (DakoCytomation, Glostrup. Denmark, K4001)	HRP anti-rabbit envision+ system (DakoCytomation, Glostrup. Denmark, K4003)
**Tertiary antibody**	Not applicable			
**Visualisation staining**	DAB, diaminobenzidine (DakoCytomation, Glostrup. Denmark, K3468)			
**Counterstaining**	Hematoxylin			

MBL, mannose-binding lectin; HPA, human platelet antigen; Tris, tris(hydroxymethyl)aminomethane; EDTA, ethylenediaminetetraacetic acid; PBS, phosphate buffered saline; BSA, bovine serum albumin; HRP 1) B2G1 was kindly provided by Dr. W. Ouwehand and Dr. C. Ghevaert, (University of Cambridge, NHS, Blood and Transplant, Cambridge, UK) B2G1 was conjugated with horseradish peroxidase (HRP), using Lightning-Link HRP Conjugation Kit (Innova Biosciences, Cambridge, UK, 701-0000), according to manufacturers' instructions. 2) Anti-s was conjugated with horseradish peroxidase (HRP), using Lightning-Link HRP Conjugation Kit (Innova Biosciences, Cambridge, UK, 701-0000), according to manufacturers' instructions.
